# Demographic and Clinical Epidemiology of Irritable Bowel Syndrome in U.S. Community Health Centers: A Cross-Sectional Analysis of the 2022–2023 National Ambulatory Medical Care Survey

**DOI:** 10.7759/cureus.87124

**Published:** 2025-07-01

**Authors:** Kingsley O Ozojide, Adaobi A Ozigbo, Kuukua K Ghartey, Okelue E Okobi, Nneka Muoghalu, Olabisi A Gbagba, Aminat D Lawal, Tinuola Andre (Fakoya), Simon Egyin, Faith C Anyanwu, Rukayat O Balogun

**Affiliations:** 1 Public Health, Nottingham Trent University, Nottingham, GBR; 2 Internal Medicine, University at Albany, State University of New York, New York, USA; 3 Accident and Emergency, Korle Bu Teaching Hospital, Accra, GHA; 4 Family Medicine, IMG Research Academy and Consulting LLC, Homestead, USA; 5 Family Medicine, Larkin Community Hospital Palm Springs Campus, Miami, USA; 6 Family Medicine, Lakeside Medical Center, Belle Glade, USA; 7 Public Health, University of Liverpool, School of Tropical Medicine, Liverpool, GBR; 8 General Medicine, University College Hospital, Ibadan, NGA; 9 Family Medicine, University of Ghana Medical School, Accra, GHA; 10 Public Health, University of New Haven, West Haven, USA; 11 Internal Medicine, Piedmont Athens Regional, Athens, USA; 12 Medicine, University College Hospital, Ibadan, NGA; 13 Health Security, Johns Hopkins Bloomberg School of Public Health, Baltimore, USA; 14 Community Health and Primary Health Care, Lagos State University Teaching Hospital, Lagos, NGA; 15 Family Medicine, National Health Service (NHS) England North East, Sunderland, GBR

**Keywords:** anxiety, community health centers, comorbidity, gerd, hypertension, irritable bowel syndrome, namcs, race, sex

## Abstract

Background

Irritable bowel syndrome (IBS) is a prevalent, burdensome gastrointestinal disorder frequently encountered in primary care. However, its demographic and comorbidity patterns in community health center (CHC) settings remain understudied.

Objective

To examine the demographic and comorbidity-related factors associated with IBS visits among U.S. community health center patients using nationally representative data.

Methods

A retrospective, cross-sectional analysis was conducted using the 2022-2023 National Ambulatory Medical Care Survey (NAMCS). Survey-weighted logistic regression was used to assess associations between IBS diagnosis and demographic factors (age, sex, race/ethnicity) and comorbidities (anxiety, gastroesophageal reflux disease (GERD), migraine, chronic pain, diabetes, and hypertension).

Results

Female sex (adjusted odds ratio (aOR)=1.84), comorbid anxiety (aOR=1.69), and GERD (aOR=1.77) were significantly associated with higher odds of IBS. Race and ethnicity also showed significant variation, with Black and "Other" race patients having lower odds of IBS. Interestingly, hypertension was inversely associated with IBS (aOR=0.49), suggesting potential differences in clinical presentation or comorbidity clustering.

Conclusion

IBS in CHC settings is more prevalent among women and those with anxiety or GERD. At the same time, racial disparities and an inverse relationship with hypertension highlight the need for nuanced, patient-centered approaches in primary care.

## Introduction

Irritable bowel syndrome (IBS) is a particularly prevalent functional gastrointestinal disorder that affects the quality of life and involves resources such as time and money on healthcare across the United States. IBS affects roughly 10%-15% of adults and is characterized by chronic abdominal pain and alteration of bowel habits in the absence of underlying organic pathology. However, prevalence estimates often differ greatly owing to the population studied or the criteria used for diagnosis [1]. Non-life-threatening as it may be, IBS results in an enormous burden on individuals and health systems, first in terms of loss of productivity due to missed workdays and increased absenteeism [2]. It also has frequent unscheduled physician visits and high consumption of diagnostic procedures, pharmacological treatments, and even over-the-counter medications [3-5]. The economic burden of IBS, as observed in primary care and community medicine, is more acute, as this is the first treatment setting where patients present their symptoms [6].

Epidemiological research has shown IBS to be a condition that disproportionately affects women and people below the age of 50. It also affects those with some psychosocial or pain-related comorbidity [7]. Chronic pain, depression, anxiety, and migraines are just a few of the many conditions that are considered to be associated with IBS [8]. However, the association between IBS and comorbidities, particularly in diverse populations treated in community health centers, remains poorly investigated in the current literature [9,10].

Race and ethnicity influence the presentation and diagnosis of IBS, although the evidence is not uniform. The different cultures within a society have diverse reporting patterns of symptoms, utilization of care, and literacy levels about healthcare [11]. They even have the level of care and diagnostic procedures that have differences, which add to the gap [12]. Moreover, chronic conditions like type 2 diabetes are now accepted to have some form of relationship with the dysregulation of gastrointestinal processes and could interact with the symptoms of IBS in multifaceted ways [13,14]. Demographics combined with other comorbid diagnoses is an important factor that needs deeper understanding as it impacts the perception and treatment of IBS across different populations [15].

Community health centers (CHCs) represent a major entry point for primary care services and are a special boon for medical-care deserts and poorer segments of the population. These centers attend to the patients who are often ignored in big epidemiologic studies, such as racial and ethnic groups, non-English speakers, and the uninsured [16,17]. Hence, CHCs serve as an important window into how IBS is clinically presented, managed, and treated in different population groups across the United States [18].

Despite the elevated frequency and healthcare burden of IBS, little national information is available detailing its clinical and demographic profile in CHCs. Prior research has systematically utilized insurance data or academic health centers, which might not reflect populations receiving care in safety-net settings [19]. Data from the 2022 National Ambulatory Medical Care Survey (NAMCS), a vast database with a wide representation of community health visits across the United States, was used [20,21]. The objective of this study is to (1) quantify associations between IBS diagnosis and demographic factors (age, sex, race/ethnicity) in U.S. community health center visits; (2) evaluate the prevalence and impact of key comorbidities (anxiety, gastroesophageal reflux disease (GERD), migraine, chronic pain, hypertension, and diabetes) on IBS‐related visits; and (3) explore potential disparities in IBS diagnosis across subgroups and identify targets for future interventions.

## Materials and methods

Study design and data source

This study employed a cross-sectional design utilizing pooled data from the 2022 and 2023 cycles of the NAMCS [21]. Conducted annually by the National Center for Health Statistics (NCHS), NAMCS is a nationally representative survey that captures visit-level information from non-federal, office-based physicians and CHCs across the United States. The survey uses a multistage probability sampling design that incorporates stratification, clustering, and unequal probabilities of selection to ensure an accurate national representation of ambulatory care services. The dataset includes comprehensive data on patient demographics, diagnoses, visit characteristics, and treatments administered during medical encounters. Visits are selected by geographic primary sampling unit (PSU), then by provider, and then by random sampling of patient encounters. Diagnoses are abstracted by trained staff from medical records during a standardized one-week reporting period per provider.

Inclusion and exclusion criteria

The analytic sample was restricted to adult patient visits involving individuals aged 18 years and older. While age was not significantly associated with IBS in multivariable models, exploratory analyses suggest older adults may underreport IBS symptoms or attribute them to other chronic conditions. Future research should investigate these age-related reporting patterns and consider longitudinal designs to clarify temporal relationships and underdiagnosis in older populations.

Visits involving patients younger than 18 years were excluded. Additionally, records with missing or inapplicable responses for key variables, specifically those coded as -9 in the NAMCS dataset, were removed. In total, 312 visits (12.5% of the original pool) were excluded due to missing demographic or diagnostic data, leaving a final unweighted sample of 2,189 adult visits from the two years. While this approach helps ensure data completeness, we acknowledge that exclusion of records with missing data may limit the generalizability of our findings and could introduce selection bias; future research should explore imputation methods or sensitivity analyses to assess the impact of these exclusions.

Survey weighting and analytic sample

To produce nationally representative estimates and account for the survey’s complex sampling design, we applied NAMCS-provided patient visit weights via Stata’s svyset (PSU, strata, weight) commands in Stata 18 (Stata Corp, College Station, TX); weights account for nonresponse and post-stratification. Missing continuous variables (<1%) were imputed via mean substitution; categorical missingness was handled via listwise deletion. These weights adjust for selection probability, nonresponse, and post-stratification. After applying the weights, the analytic sample represented an estimated 600,878 adult ambulatory care visits in the United States from 2022 to 2023 [21]. This approach allows inferences to the broader U.S. adult ambulatory population, not just the sampled visits.

Variables

Outcome Variable

The primary outcome was the presence of IBS, identified by the International Classification of Diseases, Tenth Revision, Clinical Modification (ICD-10-CM) diagnosis codes documented during the patient visit, K58.x; anxiety by F41.x; GERD by K21.x; migraine by G43.x; chronic pain syndrome by G89.4; hypertension by I10; and diabetes mellitus by E11.x. A binary variable, has_ibs, was created and coded as 1 if IBS was recorded and 0 otherwise.

Independent Variable

Independent variables included both demographic and clinical characteristics.

Demographics

Sex (men/women), continuous age, and categorized age groups (18-34, 35-49, and ≥65 years) were recorded. These cut-points were chosen to align with prior IBS epidemiology studies, which often demonstrate the highest prevalence under age 50 and a notable drop after retirement age. We did not subdivide the 50-65 interval to preserve sufficient sample size per stratum and avoid unstable estimates; however, future work could explore finer age granularity, particularly within the 50-65 bracket, to detect more nuanced trends.

Clinical Comorbidities

Binary indicators (present=1, absent=0) for the following conditions: anxiety disorders, migraine, chronic pain syndrome, hypertension, GERD, diabetes mellitus.

Statistical analysis

All statistical analyses were conducted in Stata 18, incorporating the survey design using the `svy’ commands to account for stratification and clustering. Descriptive statistics were reported as weighted means and standard deviations for continuous variables and weighted proportions for categorical variables. Differences in categorical variables by IBS status were assessed using design-adjusted chi-square (F) tests, while age differences were evaluated using survey-adjusted t-tests. To examine independent associations between patient characteristics and IBS, we performed multivariable logistic regression using the svy: logistic command. The model included all demographic and clinical covariates simultaneously. The results are presented as adjusted odds ratios (aORs) with 95% confidence intervals and associated p-values. Statistical significance was determined at a 5% level of significance.

Ethical considerations

This study was based on a secondary analysis of publicly available, de-identified data from the 2022-2023 NAMCS, conducted by NCHS. According to U.S. federal guidelines (45 CFR 46.104(d)(4)), this research was deemed exempt from institutional review board (IRB) oversight, as it involved existing data that cannot be linked to individual identities. The appropriate federal oversight bodies approved the original NAMCS data collection procedures, and no additional informed consent or ethical review was required for this secondary analysis. Though exempt from IRB, NAMCS data collection follows Health Insurance Portability and Accountability Act (HIPAA)-equivalent confidentiality standards; no individual identifiers are released.

Although our findings highlight significant racial and ethnic disparities in IBS diagnosis, additional sociocultural factors, such as differences in symptom reporting language, health literacy, cultural stigma around gastrointestinal complaints, and structural barriers to specialist referral, may underlie these patterns. A more in-depth qualitative or mixed-methods exploration of these dimensions would be valuable to contextualize and mitigate disparities in community health center settings.

## Results

Descriptive statistics and bivariate associations

Table 1 presents the weighted distribution of visits with and without a diagnosis of IBS across key demographic characteristics. Among the estimated 600,878 adult visits recorded in the 2022-2023 NAMCS data, approximately 7% (n=42,667) were associated with an IBS diagnosis.

**Table 1:** Weighted Distribution of Irritable Bowel Syndrome (IBS) Visits by Demographic Characteristics, NAMCS 2022–2023 (N=600,878 Visits) Note: Age is reported in years as mean±SD. Proportions are weighted and shown as percentages. Statistical significance was determined at p<0.05. - entries refer to intentionally left blank.

Variable Name	Description	IBS (N=42,667)	Non-IBS (N=558,211)	T-test	Chi-square	P-value
Age	Patient age in years	55.75±13.95	59.34±13.42	3.24	-	0.003
Sex	Patient sex	-	-	-	10.06	<0.001
Male	-	6050 (4%)	142759 (96%)	-	-	-
Female	-	36617 (8%)	415451 (92%)	-	-	-
Racereth	The patient's race and Hispanic ethnicity	-	-	-	25.07	0.05
Non-Hispanic White	-	28928 (10%)	265551(90%)	-	-	-
Non-Hispanic Black	-	4546 (4%)	113450 (96%)	-	-	-
Hispanic	-	7751 (5%)	134773 (95%)	-	-	-
Other race	-	1442 (3%)	44438 (97%)	-	-	-
Age group	Patient age group	-	-	-	17.75	0.03
18-44 years	-	11168 (12%)	79106 (88%)	-	-	-
45-64 years	-	20560 (7%)	283604 (93%)	-	-	-
65 years or more	-	10940 (5%)	195502 (95%)	-	-	-

Age

Patients with IBS were significantly younger on average (mean=55.75 years, SD=13.95) compared to those without IBS (mean=59.34 years, SD=13.42), with a statistically significant difference (t=3.24, p=0.003). This suggests a modest but notable tendency for IBS diagnoses to be more prevalent in relatively younger adult populations.

Sex

There was a clear sex disparity in IBS-related visits. Women accounted for 36,617 (8%) of IBS visits, while men comprised only 6,050 (4%). This difference was statistically significant (χ²=10.06, p<0.001), indicating that women were substantially more likely than men to have visits related to IBS.

Race/Ethnicity

IBS prevalence varied across racial and ethnic groups, with the highest proportion observed among non-Hispanic White individuals, 28,928 (10%), followed by Hispanic, 7,751 (5%); non-Hispanic Black, 4,546 (4%); and other racial groups, 1,442 (3%). The association between race/ethnicity and IBS diagnosis was statistically significant (χ²=25.07, p=0.05), highlighting potential racial/ethnic disparities in either the diagnosis or presentation of IBS in ambulatory care.

Age Groups

IBS was more frequently observed among adults aged 18-44 years, 11,168 (12%), and 45-64 years, 20,560 (7%), compared to those aged 65 and older, 10,940 (5%). The difference across age groups was statistically significant (χ²=17.75, p=0.03), supporting the interpretation that IBS is disproportionately diagnosed among younger and middle-aged adults in community health center settings.

Comorbidity characteristics and bivariate associations

Table 2 outlines the weighted distribution of IBS-related visits according to comorbid clinical conditions.

**Table 2:** Weighted Distribution of Irritable Bowel Syndrome (IBS) Visits by Comorbidity Characteristics, NAMCS 2022–2023 (N = 600,878 Visits) Note: Each comorbidity is coded as a binary variable (0=absent, 1=present). Percentages are survey-weighted. Statistical significance was assessed at p<0.05. - means intentionally left blank.

Variable Name/Values	IBS (N=42,667)	Non-IBS (N=558,211)	Chi-square	P-value
Anxiety	-	-	20.18	0.002
0	13296 (5%)	278227 (95%)	-	-
1	29372 (9%)	279984 (91%)	-	-
Diabetes	-	-	4.44	0.17
0	26244 (8%)	294509 (92%)	-	-
1	16424 (6%)	263702 (94%)	-	-
Migraine	-	-	3.29	0.17
0	31564 (7%)	446908 (93%)	-	-
1	11103 (9%)	111302 (91%)	-	-
Hypertension (HTN)	-	-	26.74	0.001
0	18770 (12%)	139717 (88%)	-	-
1	23897 (5%)	418495(95%)	-	-
Chronic Pain Syndrome	-	-	1.64	0.36
0	36355 (7%)	494682 (93%)	-	-
1	6313 (9%)	63529 (91%)	-	-
Gastroesophageal reflux disease (GERD)	-	-	15.88	0.002
0	13413 (5%)	267860 (95%)	-	-
1	29254 (9%)	290351 (91%)	-	-

Anxiety Disorders

IBS visits were significantly more common among individuals with anxiety (29,372, 9%), compared to those without anxiety (13,296, 5%), with a strong statistical association (χ²=20.18, p=0.002). This finding reinforces the well-documented link between IBS and psychological comorbidities, particularly anxiety.

Hypertension

Interestingly, IBS was more prevalent among patients without hypertension (18,770, 12%) than those with hypertension (23,897, 5%), a statistically significant difference (χ²=26.74, p=0.001). This inverse relationship may reflect differences in clinical profiles or healthcare-seeking behaviors among patients with cardiovascular comorbidities versus those presenting primarily with gastrointestinal symptoms.

Gastroesophageal Reflux Disease

A significant association was found between GERD and IBS. Patients with GERD had a higher prevalence of IBS (29,254, 9%) compared to those without GERD (13,413, 5%) (χ²=15.88, p=0.002), suggesting a potential overlap or shared pathophysiological mechanisms between the two conditions.

Diabetes, Migraine, and Chronic Pain Syndrome

While IBS prevalence appeared slightly higher among patients with these conditions (e.g., 11,103 (9%) among those with migraines vs. 31,564 (7%) without), none of the associations reached statistical significance (all p>0.05). This suggests that while these comorbidities may co-occur with IBS, their presence alone does not independently differentiate IBS from non-IBS visits at the bivariate level.

Multivariable logistic regression

Table 3 presents the results of a multivariable, survey-weighted logistic regression model identifying factors independently associated with IBS diagnosis after controlling for demographic and clinical characteristics.

**Table 3:** Multivariable Survey-Weighted Logistic Regression of Factors Associated with Irritable Bowel Syndrome (IBS) Among U.S. Community Health Center Visits, NAMCS 2022–2023 (N=600,878 Visits) Note: Odds ratios (ORs) were derived using survey-weighted logistic regression to account for the complex sampling design. P-values <0.05 were considered statistically significant. Reference categories: Male (sex), Non-Hispanic White (race/ethnicity), and age 18–34 (age group). - means intentionally left blank

Variable	Odds Ratio (OR)	St. Error	95% CI (Lower-Upper)	p-value
Sex	-	-	-	-
Female (vs. Male)	1.84	0.38	1.20-2.83	0.01
Race/Ethnicity	-	-	-	-
Black (vs. White)	0.46	0.17	0.21-1.01	0.05
Hispanic	0.68	0.32	0.26-1.81	0.43
Other	0.39	0.16	0.17-0.89	0.03
Age Group	-	-	-	-
35-49 (vs. 18-34)	0.69	0.25	0.33-1.46	0.32
65+	0.65	0.22	0.32-1.32	0.22
Comorbidities	-	-	-	-
Anxiety	1.69	0.36	1.10-2.61	0.02
Migraine	0.84	0.25	0.45-1.56	0.57
Chronic Pain	1.08	0.34	0.56-2.06	0.82
Hypertension	0.49	0.12	0.30-0.81	0.01
Gastroesophageal Reflux Disease (GERD)	1.77	0.36	1.16-2.68	0.01
Diabetes	1.03	0.26	0.61-1.73	0.91

**Figure 1:**
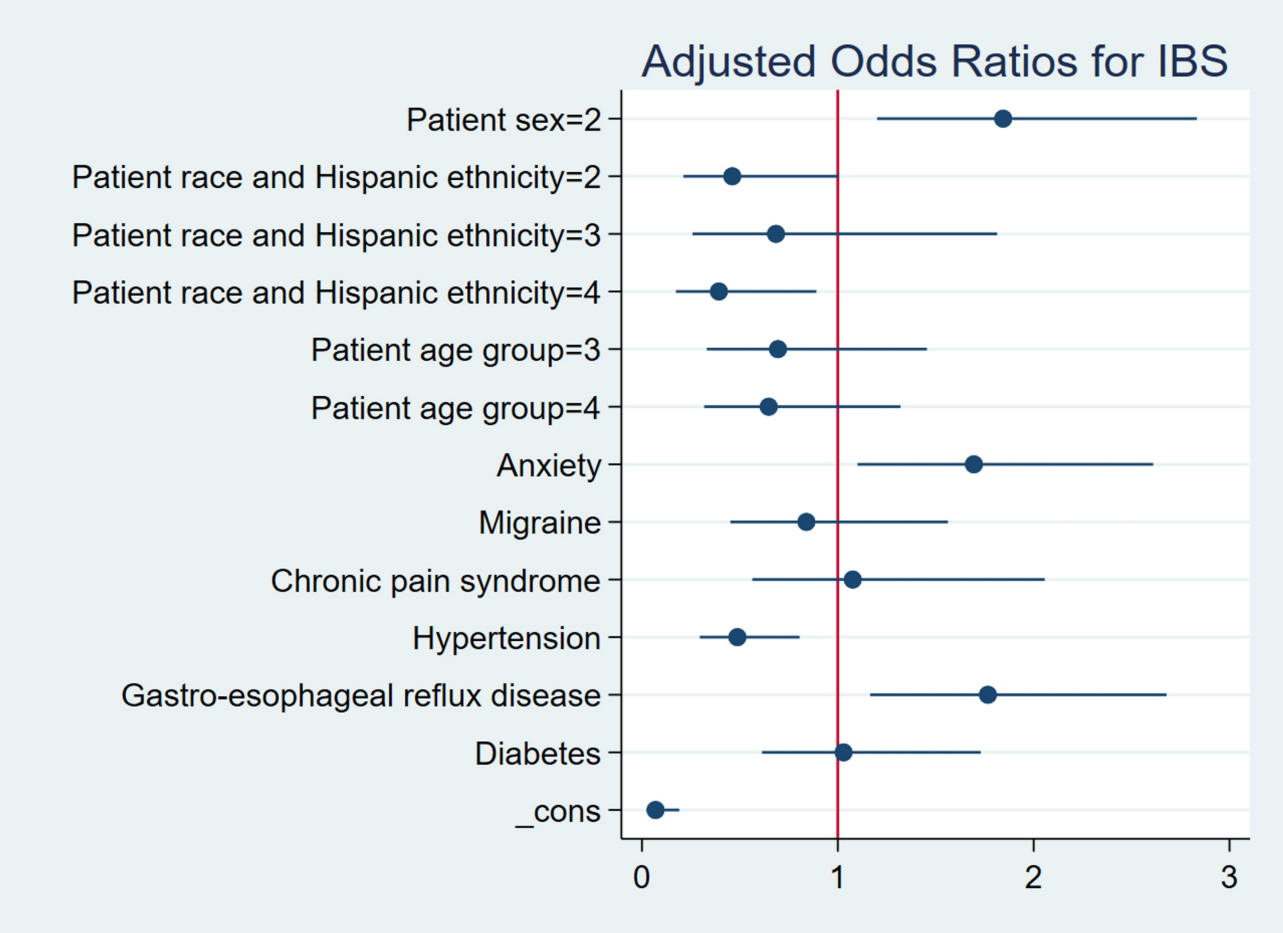
Forest Plot of Adjusted Odds Ratios (ORs) and 95% Confidence Intervals for Factors Associated with Irritable Bowel Syndrome (IBS) Among U.S. Community Health Center Visits, NAMCS 2022–2023 (N = 600,878 Visits)

**Figure 2:**
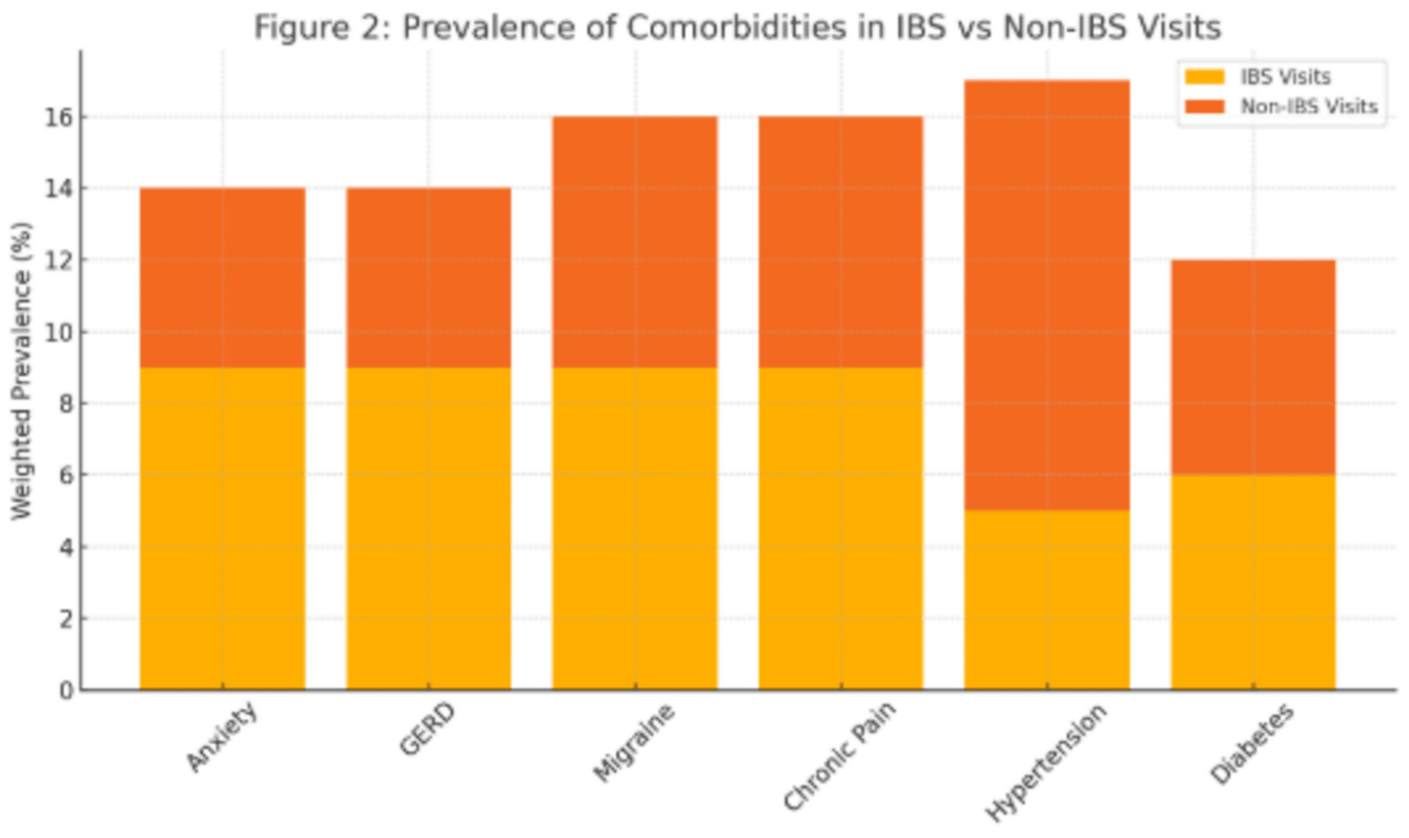
Prevalence of Comorbidities in IBS vs. Non-IBS Visits

**Figure 3:**
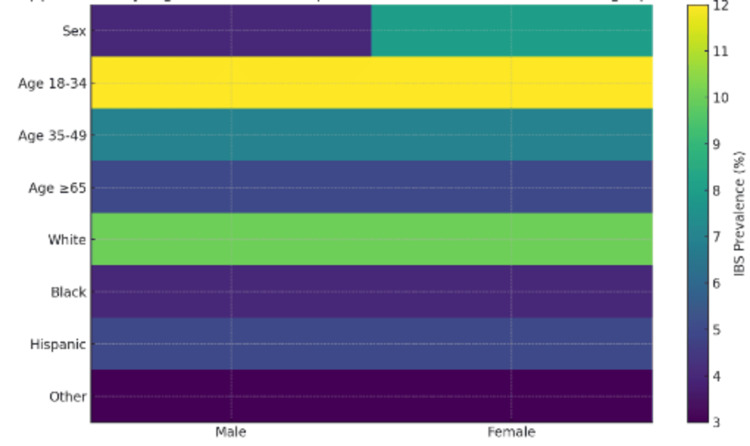
Heat Map of IBS Prevalence Across Demographic Strata

Multivariable logistic regression analysis in Table 2 revealed that several demographic and clinical factors were significantly associated with an IBS diagnosis. Female sex was associated with significantly higher odds of IBS compared to male patients (aOR=1.84, 95% CI: 1.20-2.83, p=0.01), a finding consistent with the existing literature that attributes this disparity to hormonal influences, psychosocial factors, and differences in healthcare-seeking behavior. Regarding race and ethnicity, non-Hispanic Black individuals had lower odds of IBS diagnosis compared to non-Hispanic Whites (aOR=0.46, 95% CI: 0.21-1.01, p=0.05), with individuals classified as "Other" also demonstrating significantly reduced odds (aOR=0.39, 95% CI: 0.17-0.89, p=0.03). Hispanic ethnicity was not significantly associated with IBS (aOR=0.68, 95% CI: 0.26-1.81, p=0.43). These patterns suggest potential disparities in the diagnosis or access to care among racial and ethnic minority groups. In terms of age, no significant differences in IBS odds were observed across age groups relative to the 18-34 reference group, though a non-significant trend suggested lower odds in older adults (ages 35-49: aOR=0.69; 65 and older: aOR=0.65). Clinically, anxiety was significantly associated with increased odds of IBS (aOR=1.69, 95% CI: 1.10-2.61, p=0.02), reinforcing the psychological component often linked with the condition. Similarly, GERD was associated with higher odds of IBS (aOR=1.77, 95% CI: 1.16-2.68, p=0.01), reflecting possible pathophysiological overlap between gastrointestinal disorders. In contrast, hypertension was inversely related to IBS (aOR=0.49, 95% CI: 0.30-0.81, p=0.01), potentially indicating differences in clinical priorities or comorbidity clustering. Migraine, chronic pain syndrome, and diabetes were not significantly associated with IBS after adjusting for covariates, suggesting that confounding factors may explain their observed bivariate associations. Figure 1 illustrates the adjusted odds ratios and 95% confidence intervals from the multivariable logistic regression model, highlighting factors independently associated with IBS among U.S. community health center visits. In addition, Figure 2 displays the survey-weighted prevalence of clinical comorbidities in IBS versus non-IBS visits, highlighting the relative overrepresentation of anxiety and GERD among IBS encounters. Figure 3 uses a heat map to visualize IBS prevalence across demographic strata, sex, age group, and race/ethnicity, providing an at-a-glance view of the gradients observed in our analyses.

The forest plot in Figure 1 presents aORs from a logistic regression model examining factors associated with IBS. Notably, female patients had significantly higher odds of an IBS diagnosis compared to male patients, while patients with anxiety and GERD also showed increased odds, indicating strong associations with these conditions. In contrast, hypertension was significantly associated with lower odds of IBS. Additionally, individuals in the "Other" race/ethnicity category had reduced odds compared to non-Hispanic Whites. Other variables, including age group, migraine, chronic pain, and diabetes, did not show statistically significant associations, as their confidence intervals included the null value (OR=1).

## Discussion

This study offers an in-depth analysis of demographic and clinical characteristics associated with IBS in the context of U.S. CHCs, drawing on nationally representative data from the 2022 NAMCS [21]. Our findings contribute to a more comprehensive understanding of IBS presentations in underserved populations and highlight several important trends about sex, race/ethnicity, age, and comorbid diagnoses.

Consistent with prior literature, female sex emerged as a strong and independent predictor of IBS diagnosis. Women had nearly twice the odds of an IBS-related visit compared to men, even after controlling for other variables. This aligns with the existing epidemiologic studies that suggest sex hormones, visceral hypersensitivity, and differential care-seeking behavior may influence both the experience and reporting of IBS symptoms among women; in particular, estrogen and progesterone are associated with increased visceral pain sensitivity and altered gut motility [22]. These hormones also influence gastrointestinal microbiota composition and immune responses, further contributing to symptom manifestation. Additionally, societal pressures on women to maintain a certain appearance, such as staying thin and well-groomed, can act as chronic stressors, potentially exacerbating IBS symptoms. Conversely, men may face stigma associated with IBS, as it is often perceived as a predominantly female condition [23]. The observed sex disparity emphasizes the need for clinicians to adopt a gender-informed approach in the diagnosis and management of IBS, especially in primary care and safety-net settings where symptoms may otherwise be underappreciated.

Racial and ethnic disparities in IBS diagnosis were evident and persisted in multivariable analysis. Non-Hispanic Black patients and those in the "Other" racial category had significantly lower odds of receiving an IBS diagnosis compared to non-Hispanic White patients. These differences may reflect a combination of cultural factors, health literacy, provider bias, and systemic inequities in healthcare delivery. Prior studies have noted that minorities may present with different symptom language or thresholds for seeking care, potentially contributing to the under-recognition of functional disorders like IBS. Additionally, racial and ethnic minorities often encounter structural barriers to diagnostic procedures, including a lack of continuity of care, language barriers, or lower referral rates for gastroenterological evaluation.

The finding that non-Hispanic Black individuals and those categorized as “Other” races have significantly lower odds of receiving an IBS diagnosis aligns with the existing literature on racial and ethnic disparities in gastrointestinal healthcare. Prior research indicates that while Black Americans report IBS-like symptoms at rates comparable to, or even higher than, White Americans in community surveys, they are less likely to receive a formal diagnosis. This suggests underdiagnosis rather than a true difference in prevalence [22,24]. Several factors may contribute to this pattern, including differences in symptom reporting, varying levels of trust in the healthcare system, and disparities in access to specialists or diagnostic testing [23-26]. Additional studies have found that racial and ethnic minorities, particularly Black and Asian individuals, are less likely to undergo procedures such as colonoscopies or receive referrals to gastroenterologists [25,27]. These insights support the interpretation that the disparities observed in IBS diagnosis are likely driven by broader systemic issues in healthcare delivery, as well as cultural and communication barriers between patients and providers.

Interestingly, age was not a statistically significant factor in multivariable models, despite showing trends in the bivariate analysis. While IBS was more frequently observed in younger patients in raw comparisons, these age effects were attenuated after adjusting for sex, race/ethnicity, and comorbidities. This may suggest that age-related differences are mediated through other clinical or behavioral variables. Still, the observation that older adults were less likely to have IBS-related visits remains clinically relevant, as symptoms may be overlooked or attributed to other chronic conditions in this population.

Our study’s finding that IBS is more prevalent among relatively younger adults aligns with broader patterns observed in global epidemiological research. A seminal meta-analysis by Lovell and Ford, which examined data from over 260,000 individuals across 80 study populations, found a higher prevalence of IBS among adults under the age of 50, indicating a potential age-related susceptibility to the disorder [28]. Similarly, the Trøndelag Health Study (HUNT) conducted in Norway reported the highest prevalence of IBS among individuals younger than 40, with a marked decline in prevalence with increasing age [29].

Several interconnected biological and psychosocial mechanisms may explain this trend. Younger adults are more likely to experience gastrointestinal infections, which are a significant risk factor for developing post-infectious IBS [30,31]. Moreover, psychological conditions such as anxiety, depression, and stress, more common in younger age groups, have been shown to exacerbate IBS symptoms through disruption of the gut-brain axis [29,32]. Importantly, the lower prevalence reported in older adults may not necessarily indicate a true reduction in IBS incidence. Instead, it could reflect diagnostic overshadowing by other chronic conditions or a decreased likelihood of older individuals seeking medical attention for functional gastrointestinal symptoms [28,32]. These findings suggest that the age-related distribution of IBS prevalence observed in our study is likely influenced by a combination of underlying pathophysiological factors and age-dependent differences in healthcare-seeking behavior and symptom attribution.

Among the comorbidities examined, anxiety and GERD were the only conditions significantly associated with IBS after adjustment. The strong link between anxiety and IBS is well-established and reflects the central role of the brain-gut axis in functional gastrointestinal disorders. This finding reinforces the importance of integrated care approaches that incorporate behavioral health into gastrointestinal care, particularly in primary care settings like CHCs, where mental health resources may be limited.

Similarly, the association between GERD and IBS supports the existing literature, suggesting an overlap in symptomatology and shared pathophysiological mechanisms such as visceral hypersensitivity and gastrointestinal motility disorders. These patients may benefit from coordinated evaluation and management strategies that consider both upper and lower gastrointestinal (GI) symptoms concurrently.

Conversely, hypertension was negatively associated with IBS diagnosis, a somewhat unexpected finding. One possible explanation is that patients with chronic cardiovascular conditions like hypertension may have different visit priorities, and functional gastrointestinal symptoms may be underreported or underexplored during routine care. Alternatively, the medication regimens and clinical monitoring of hypertensive patients may modulate GI symptoms in ways not fully captured in this analysis.

Finally, while migraine, chronic pain syndrome, and diabetes were associated with IBS in our bivariate analysis, they were not significant in the adjusted model. This suggests that these conditions may be indirectly related to IBS through shared psychosocial or physiological pathways, such as anxiety or somatization, rather than exerting an independent effect. For instance, in their study, Bakshi et al. reported that it was often challenging to distinguish pain from other gastrointestinal inflammatory symptomatology that negatively affects the psychosocial and physical abilities of the patients; hence, the observed dissociation warrants independent and rightful management of pain to improve the quality of life of IBD patients [33].

Our findings carry practical relevance for CHC operations and treatment strategies. First, the strong associations with anxiety and GERD underscore the value of embedding behavioral health screening and coordinated gastroenterology referrals into routine CHC workflows. Integrating mental health professionals or training primary care staff in brief cognitive-behavioral techniques may help address the psychosocial contributors to IBS in real time. Second, the identification of sex and racial/ethnic disparities suggests that CHCs should implement culturally sensitive education materials, leverage language-concordant navigators, and adopt standardized symptom-checklists to reduce diagnostic bias. Lastly, establishing multidisciplinary care pathways, linking dietitians, mental health counselors, and primary care providers, can streamline treatment, improve symptom control, and enhance patient satisfaction among CHC populations.

Limitations of the study

This study has several limitations worth noting. First, the analysis relied on secondary data from the NAMCS, which inherently included missing or incomplete information. Records with missing values on key demographic or diagnostic variables had to be excluded, which may have introduced selection bias and reduced the representativeness of the sample. Moreover, because NAMCS is a cross-sectional, visit-level survey, we cannot distinguish between multiple visits by the same patient or capture longitudinal symptom trajectories. Second, the cross-sectional nature of the data limits causal inference; associations observed cannot confirm temporal relationships between IBS and comorbidities. Third, diagnostic misclassification is possible: IBS identification depended solely on ICD-10 codes recorded at the visit, without access to clinical details or Rome criteria verification, and comorbidity coding may similarly underreport conditions managed outside the CHC. Fourth, unmeasured confounding, such as socioeconomic status, diet, or medication use, could bias the estimated associations. Fifth, NAMCS does not capture IBS severity, symptom chronicity, or treatment adherence, limiting our ability to relate epidemiologic patterns to clinical outcomes. Finally, although CHCs serve diverse, underserved populations, findings may not generalize to patients in private practices, hospital outpatient clinics, or non-U.S. settings.

## Conclusions

In conclusion, this study found that female sex, anxiety, and GERD were significantly associated with increased odds of IBS-related visits in the U.S. CHCs. Conversely, hypertension was inversely associated with IBS, suggesting possible differences in clinical priorities or comorbidity clustering that may influence diagnosis or reporting patterns. Additionally, racial and ethnic minority groups were less likely to receive an IBS diagnosis, highlighting potential disparities in symptom recognition or access to care. No significant associations were observed with age, migraine, chronic pain, or diabetes after adjustment. These findings emphasize the multifactorial nature of IBS diagnosis and the importance of addressing demographic and clinical context in community-based care settings. Overall, this study makes important contributions to the knowledge base around IBS in community health settings, reinforcing the need for ongoing research, targeted interventions, and improved awareness of IBS across diverse patient demographics. By addressing the outlined weaknesses and integrating suggested improvements, the study can positively impact clinical practice and enhance future research endeavors in this vital area of healthcare.
